# On the Sanative Powers of Lime Water in Diabetes

**Published:** 1802-01

**Authors:** A. J. Schutz


[ To be continued. ]

---

### *On the fanative Powers of Lime Water in Diabetes, by* Dr. A. J. Schutz.

THE nature of diabetes is not yet fo clearly afcertained as to enable us to eftablifh a fure, and as it were, fcientific method of treatment for the cure of this remarkable difeafe; but we may venture to fay, that the practice employed for that purpofe is entirely empirical, and the general indication of cure merely confined to the preceding debilitating caufes, as cold, fright, fatigue, &c. according to which roborant, aftringent, diaphoretic, antifpafmodic, anti-acid, &c. remedies were ufed, though they proved very rarely fuccefsful in performing a lafting cure. The only, or at leaft fureft, way of arriving at a radical mode of treatment in that difeafe, is by accurate obfervations, by means of which we may be led by conjectures, as well as by farther experience, to know the action of a certain clafs of remedies in the different modifications in which diabetes occurs. In the year 1789, I had an opportunity of obferving five cafes of diabetes diabetes of three different kinds, viz. diabetes aquofus, mellitus, and hyftericus, which were treated in the Clinical fchool at Pavia, then conducted by J. P. Frank, one of the moft celebrated practitioners of the Continent. Againft the two firft fpecies, alumen, pulvis Doveri, Dr. Griffith's myrrh and fteel mixture, and the tinctura cantharidum had fome effect, which, however, was of fhort duration; and though the diabetes hyftericus was removed by Griffith's mixture, yet the nervous fymptoms, which attended it, continued for a confiderable time. The prefcription for that mixture was thus changed: ℞. Sal. abfynthii, dr. j. folve in aqu. menth. piperit. unc. vj. adde fal. mart. factit. gr. xij. aqu. cinamom. unc. fs. M. D. S. A table fpoonfull to be taken every two hours. Mr. Jofeph Frank fucceeded in curing in the Clinical Difpenfary at Pavia, a diabetes aquofus with mercurial frictions and internal roborant medicines in the fpace of fixty-two days.

Having premifed thefe few remarks on the mode of treatment of diabetes, as far as I had myfelf obferved it, I proceed to give, by the fubfequent cafes, fome obfervations on the ufe of lime-water, in a full dofe, in feveral inftances of diabetes, by which I may prefume to contribute a fhare, however fmall, towards the cure of fo interefting a difeafe.

*Case* 1. The patient was by trade a butcher, fifty years old, of a lean habit and pale complexion, who had, from his youth, too much indulged himfelf in the ufe of wine and other intemperances, which mode of living he had of late been obliged to quit through poverty. His bufinefs, which was to drive cattle to the Imperial camp, expofed him to much fatigue, and frequently in fummer time he quenched his thirft with an immoderate draught of cold water, flept often in open air during night; and in one of thefe nights he caught cold to fuch a violent degree that he felt, the next morning, the greateft febrile heat and thirft; his refpiration became difficult, and his chilled feet were fwollen, in which ftate I found him on his applying to me for affiftance; he was, befides, very coftive, and had little appetite; but what he himfelf did not fo much regard, on account of the other fymptoms, was the great quantity of urine which he difcharged, whereby his fkin was parched; and he felt a burning fenfation in the bowels and a preffure in the region of the kidneys. The pulfe flow and foft; his ftrength decreafed vifibly. I defired him to meafure the quantity of urine which he excerned, and it exceeded, in the night of June 21, ten pints, though he had purpofely, notwithftanding his great thirft, confumed no more than about one pint of fluid!!—June 22. The patient complained of a fourifh tafte; the quantity of urine paffed in twenty-four hours was about 22 pints; pulfe flow; and and only 32 ſtrokes in a minute.—June 24. He paſſed in twenty-four hours only 18 pints of urine, which looked a little bloody, exhaling a putrid ſtench; pulſe 52 in a minute.—June 25. He had again diſcharged 22 pints of urine; pulſe 54. The preſſure and ſenſation in the region of the kidney had entirely ceaſed, appetite better, but thirſt equally great. The following days the quantity of urine paſſed in twenty-four hours varied from 17 to 22 pints. The ſkin remained always dry and parched, notwithſtanding the thermometer was raiſed to 30° Reaumur; however, the patient found himſelf a little better in hot weather. After having thus convinced myſelf of the preſence and degree of diabetes, I had recourſe to the lime-water, which I got prepared from oyſter ſhells, and I ordered a wine glaſs full of it to be taken every two hours, beſides which I had volatile liniments rubbed in the lumbar region, and clyſters were applied for removing the coſtiveneſs. His thirſt increaſed at firſt, but he had hardly taken a few pints of lime-water when the quantity of urine that paſſed began conſiderably to decreaſe, not exceeding ſeven or eight pints in twenty-four hours, three days after having begun to drink the lime-water, which I, therefore, ordered to be continued to one pint a day. The ſixth day the quantity of urine paſſed in twenty-four hours was five pints. The patient was daily recovering his ſtrength; and thirteen days after the uſual quantity of only three or four pints of urine paſſed in twenty-four hours. All other ſymptoms diſappeared, while health and ſtrength returned. I diſmiſſed him, therefore, from my care, deſiring him to continue the uſe of lime-water a little longer. The patient returned to his uſual buſineſs, and has ever ſince (about eight years ago) been perfectly well.

*Case* 2. A farmer's boy, aged 19, had been ſo frightened by one of the horſes which was entruſted to his care tumbling into a ſtone pit, that he fell ſenſeleſs on the ground; and after having recovered himſelf, he felt a moſt violent thirſt, which he quenched with ſeveral draughts of cold water; from this time his health decayed viſibly, and he became very much emaciated. He applied to ſeveral quacks in the neighbourhood, by whom he was treated for three months, without any ſucceſs, becauſe none of them could diſcover the true ſcource of his malady. When I ſaw him he was extremely emaciated, and ſo weak as to be obliged to keep his bed; his ſkin was dry and parched, his belly ſhrunk, and coſtive, pulſe ſlow and full; appetite good. He complained of pains in the lumbar region, of a burning ſenſation in the bowels, and he was tormented with the moſt violent thirſt, which frequent drinking could not allay. The chief ſymptom, however, overlooked by the quacks, was was the great quantity of urine he paſſed, and which exceeded 18 pints a day: it was of a greeniſh colour, ſmelled like a fermenting liquor, and depoſited a great quantity of ſaccharine cryſtals. All this proved the caſe to be a diabetes mellitus. The patient having taken, formerly, ſtrong draſtics, and other diſagreeable remedies, could not be perſuaded to uſe any medicine, on which account I was obliged to have recourſe to the lime-water, which I prepared weak to make it more palatable to the patient. I ordered about two pints to be taken every day. He had uſed it two days, when a topical ſweat of hands and feet came on, much to his relief, though the quantity of urine paſſed in one day remained the ſame; the fourth day, however, it had decreaſed one half. Having continued the lime-water for ſome time, the patient became daily better; his ſtrength returned, his pulſe was quicker; he was able to leave his bed and to walk about in the houſe; his preternatural appetite and thirſt had conſiderably abated, and likewiſe the quantity of urine, which, however, ſtill remained ſaccharine. The patient could not be prevailed upon to continue the uſe of lime-water any farther; but fancying that nature would finiſh the cure, he was determined neither to drink the lime-water nor to keep to a proper diet. In a ſhort time the quantity of urine which he diſcharged increaſed again, from day to day; and thus lingering, he died at laſt quite emaciated. This caſe, however, proves the uſe of lime-water, as it is moſt probable the patient would have been cured had he continued it for a longer time.

*Case* 3. A man, aged 62, had been affected with diabetes mellitus for nearly three years. He was ordered to take three cups of lime-water every day, which, having uſed for ſeveral weeks, he felt himſelf very much relieved; the burning ſenſation in the bowels and regio lumborum entirely ceaſed; while, at the ſame time, his violent thirſt abated, the quantity of urine conſiderably diminiſhed, and in a month he was perfectly cured.

*Case* 4. A man, 72 years of age, had laboured under a diabetes mellitus for ſeveral years, which was attended with hectic fever, dryneſs of ſkin, and obſtinate coſtiveneſs. He began to take lime-water, and after having continued it for ſix weeks, the hectic fever diſappeared, the diabetes diminiſhed by degrees, and in the ſpace of three months he was perfectly cured.

*Historical*

